# Social exclusion: differences in neural mechanisms underlying direct versus vicarious experience

**DOI:** 10.3389/fpsyg.2024.1368214

**Published:** 2024-08-27

**Authors:** Juan Song, Tao Lian, Yunhong Zhang, Mingjing Cao, Zhibin Jiao

**Affiliations:** ^1^Faculty of Psychology, Tianjin Normal University, Tianjin, China; ^2^National Key Laboratory of Human Factor & Ergonomics, China National Institute of Standardization, Beijing, China

**Keywords:** social exclusion, observer, vicarious experience, empathy, functional near-infrared spectroscopy

## Abstract

Social exclusion stands as a source of social discord and holds substantial research value. Prior investigations on social exclusion have overlooked the interactive relationship between the excluded individuals and the observers. Hence, this study comparatively explores the neural mechanisms underlying the psychological responses of two distinct roles within the same social exclusion context. A total of 35 pairs (19 pairs of females) participated in the experiment. Within each pair, one individual assumed the role of a socially excluded participant (target), while the other acted as a social exclusion observer. Targets engaged in an online ball-passing game where controlled ball allocations to the participants created an exclusion scenario. Meanwhile, observers spectated the targets playing the game. Throughout the ball-passing activity, functional near-infrared spectroscopy (fNIRS) recorded the blood oxygen data in the prefrontal cortex (PFC) and temporoparietal junction (TPJ) of both participants. Our findings revealed varied levels of rejection sensitivity elicited by direct or observed social exclusion experiences. Additionally, distinct patterns of neural activation were observed: targets displayed conditional differences in the medial prefrontal cortex (mPFC), while male observers exhibited conditional activation differences in the mPFC, and female observers showed conditional activation differences in the right dorsolateral prefrontal cortex (dlPFC). This study juxtaposes the behavioral and neural activation variances between targets and observers within the same social context, offering a novel perspective on investigating the neural mechanisms of social exclusion.

## 1 Introduction

Social exclusion occurs when an individual’s need for belonging is obstructed by rejection from a social group (Williams, 2007, 2009). Humans naturally seek social connections, a trait essential for survival. Exclusion disrupts these connections, leading to isolation, impacting cognition, emotions, and behavior (Baumeister et al., 2002; Twenge and Campbell, 2003; Iffland et al., 2014). Excluded individuals are prone to depression, anger, and aggression, potentially causing broader societal issues. Researching the mechanisms and factors behind social exclusion is crucial for resolving conflicts and improving individual well-being.

Research on social exclusion has traditionally examined both excluded individuals and observers. Studies reveal that ostracism leads to social pain, sharing physiological foundations with physical pain (Eisenberger et al., 2003). Even brief exclusion triggers strong rejection feelings, leading to psychological responses like diminished self-esteem and reduced meaning (Williams, 2007). Exclusion can provoke aggressive behavior (Ren et al., 2018). Observers of exclusion scenarios may experience vicarious pain (Giesen and Echterhoff, 2018), with stronger guilt, anger, and sadness when witnessing marginalized groups’ exclusion compared to dominant groups (Petsnik and Vorauer, 2020). Empathy is the ability to share the emotions and sensations of others (Singer et al., 2004). There are many theoretical explanations for vicarious exclusion, but empathy theory is the only one that can simultaneously explain changes in the fundamental needs, emotions, behaviors, and neural networks of individuals experiencing vicarious exclusion (Wesselmann et al., 2009).

Research indicates that different sexes exhibit distinct responses when faced with social exclusion (Hawes et al., 2012; Seidel et al., 2013; Brown et al., 2019). In females, rejection affects various variables such as negative emotions, control, sadness, anger, disgust, and happiness, whereas rejected males tend to display more anger and a heightened need for belongingness, control, and meaningful existence (Iffland et al., 2014). Additionally, a study investigating social exclusion in observers revealed gender disparities, with boys showing increased demand for control in social settings when exposed to social exclusion, whereas no such variations were evident among girls (Marinović and Träuble, 2021). Nevertheless, there’s a dearth of research on gender differences, especially regarding systematic investigations into neural response disparities among individuals of different genders experiencing social exclusion.

Prior research on the psychological and neural mechanisms of social exclusion has typically focused on either the individuals directly experiencing exclusion (‘targets’) or those observing it (‘observers’), neglecting the interaction between both. However, social exclusion involves both targets and observers, and the presence of observers can impact the targets’ psychological well-being, intensifying their pain (Hales et al., 2021). Additionally, there is a lack of studies exploring gender differences in the neural mechanisms of social exclusion.

To address these gaps, this study aims to simultaneously measure the neural activation patterns of both targets and observers, incorporating gender as a variable. Using functional near-infrared spectroscopy (fNIRS), which offers high ecological validity and good temporal resolution for studying social interactions (Ferrari and Quaresima, 2012; Pinti et al., 2020), this study will capture brain activity signals. Previous research has shown that social exclusion increases activation in the prefrontal cortex (PFC) among adolescents (Will et al., 2016) and enhances activation in the temporoparietal junction (TPJ) of observers (Tousignant et al., 2018). Given the importance of the PFC and TPJ in social exclusion, they have been selected as regions of interest (ROI) for measurement (Derntl et al., 2010; Meyer et al., 2013; Wudarczyk et al., 2015; Will et al., 2016; Radke et al., 2018; Tousignant et al., 2018; Jie et al., 2019; Yanagi et al., 2020).

Based on these considerations, we propose the following hypotheses: (1) Targets undergoing direct exclusion may exhibit stronger emotional reactions and greater neural activation in the PFC and TPJ compared to observers; (2) Males and females show differences in brain activation regions when encountering social exclusion. This study has significant theoretical implications: (1) It provides a comprehensive view by measuring neural activation in both targets and observers, revealing their psychological and neural mechanisms; (2) By including gender as a variable, it explores different brain activation patterns in males and females, aiding in understanding gender-specific neural mechanisms and supporting personalized psychological interventions.

## 2 Method

### 2.1 Participants

Using G*Power 3 software, sample size estimation based on relevant research (Hartgerink et al., 2015) revealed an effect size (d) greater than |1.4|, suggesting 18 participants would achieve a statistical power of 0.95. However, 18 participants are too few for fNIRS-based research, so we expanded the sample size. Under controlled social exclusion conditions, this study included 35 pairs of participants (19 female pairs; *M* = 20.15 years, SD = 2.37). Each pair was same-gender, unacquainted, right-handed, with normal or corrected vision, and no psychiatric history. The study was ethically approved by Tianjin Normal University’s Ethics Committee. Participants will receive a monetary compensation after the completion of the experiment.

### 2.2 Experimental design

**Figure 1:**
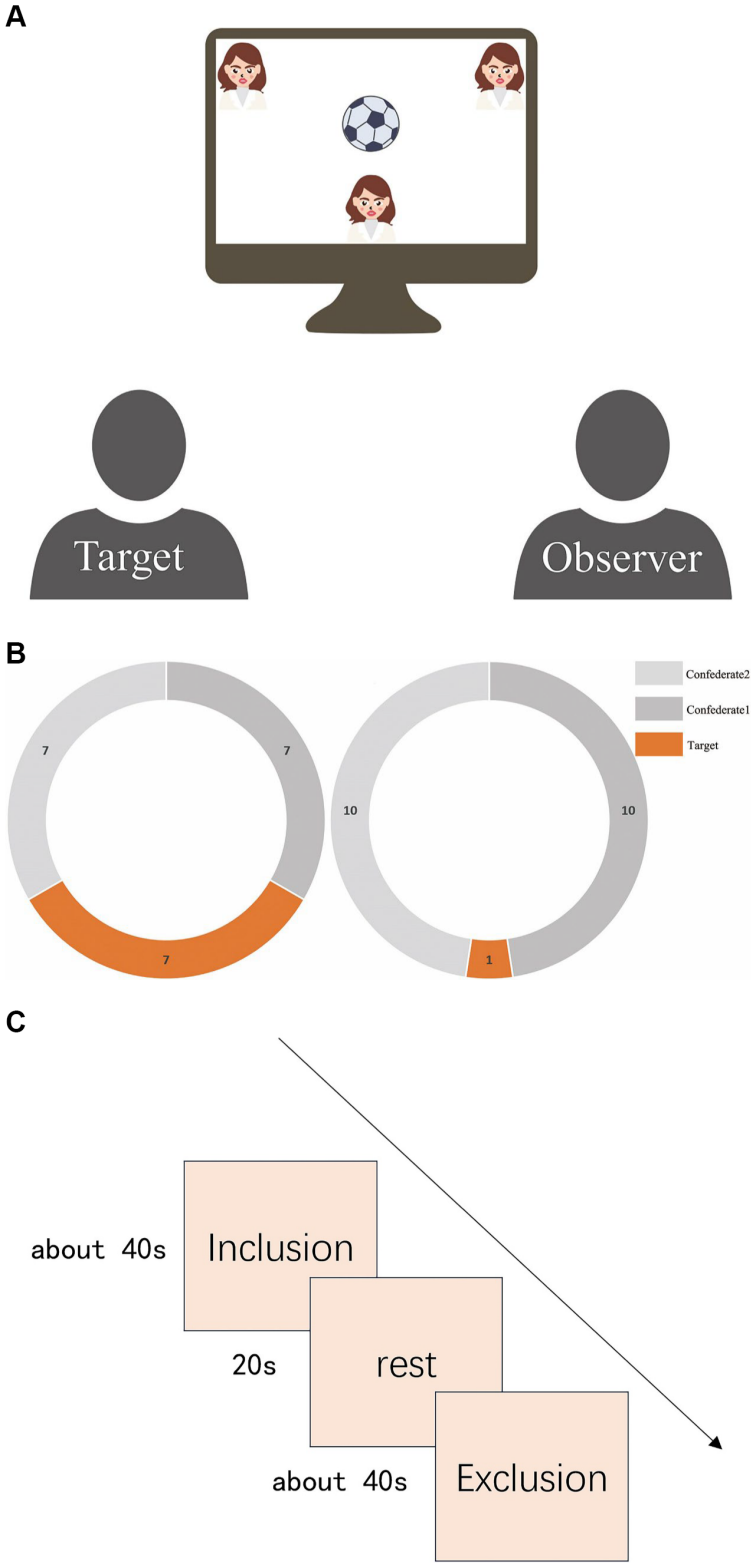
**(A)** Schematic of experimental scene. The Target is on the left, and the Observer is on the right. Both will be in the same room, watching a screen. At the top of the screen are computer-programmed confederates, but both participants will be told that these are real people participating in the experiment from another laboratory. The small figure at the bottom of the screen represents the Target. The Target will follow prompts to pass the ball by pressing keys, while the Observer will only watch this process and consider the intentions behind the ball passes. **(B)** Number of catches by targets. The left side represents the number of catches in the inclusion blocks, where the target and the other two confederates each caught the ball seven times. The right side represents the number of catches in the exclusion blocks, where the target could only catch the ball once. **(C)** Experimental procedure flowchart. There were a total of eight blocks of trials arranged in a pseudo-random distribution sequence: “inclusion, inclusion, exclusion, inclusion, exclusion, exclusion, inclusion, exclusion. “A 20-s rest interval separated each block to ensure the participants’ blood oxygen levels returned to baseline.

The experiment used the Cyberball paradigm to simulate exclusion scenarios (Williams et al., 2000; Tousignant et al., 2018). Participants were paired as ‘targets’ or ‘observers’. Targets played a ball-tossing game, while observers watched in the same lab setup. Targets were told they were playing with two others (actually computer confederates) and used keys to choose the recipients. Observers watched and considered thoughts during inclusion (equal tosses) and exclusion (one toss) conditions, arranged pseudo-randomly across eight blocks. A 20-s rest interval separated each block to ensure the participants’ blood oxygen levels returned to baseline. Furthermore, the time intervals between ball tosses by the two computer-programmed confederates were set to vary within a broad range of milliseconds. Please refer to Figure 1 for details of the experimental design.

### 2.3 Data acquisition

#### 2.3.1 Post-game assessment

After the completion of all eight blocks of the Cyberball game, the post-Cyberball assessment was used to evaluate the degree of social exclusion experienced by the targets. This scale, which ranges from 1 (indicating ‘completely absent’) to 5 (indicating ‘completely present’) on a five-point rating system, encompasses dimensions such as Belonging, Self-Esteem, Control, and Meaningful Existence. Each of these dimensions comprises five items (Williams, 2009; Cogoni et al., 2018; Ren et al., 2018). Additionally, a questionnaire on empathy was administered to measure observers’ empathy levels toward the targets. This questionnaire was based on previous studies (Fengfeng et al., 2010; Masten et al., 2011), and the relevant references have been included in the Supplementary materials for readers’ convenience.

#### 2.3.2 Empathy and rejection sensitivity assessment

We employed the Chinese revised version of the Interpersonal Reactivity Index (IRI-C) (Fengfeng et al., 2010) to gather empathy data from both targets and observers (Cronbach’s *α* = 0.75). The IRI, developed by Davis (1980, 1983), evaluates empathy across four dimensions: Emotional Understanding, Empathy Concern, Personal Distress, and Perspective Taking, using 22 items on a 5-point Likert scale. We also used the Chinese revised version of the Rejection Sensitivity Questionnaire (RSQ) (Wei, 2012) to assess participants’ sensitivity to rejection (Downey and Feldman, 1996). Participants imagined themselves in specific scenarios and rated the likelihood of receiving assistance and their apprehension about the response on a 6-point scale. The RSQ includes 18 scenarios and 36 items.

#### 2.3.3 Neural signal assessment

**Figure 2:**
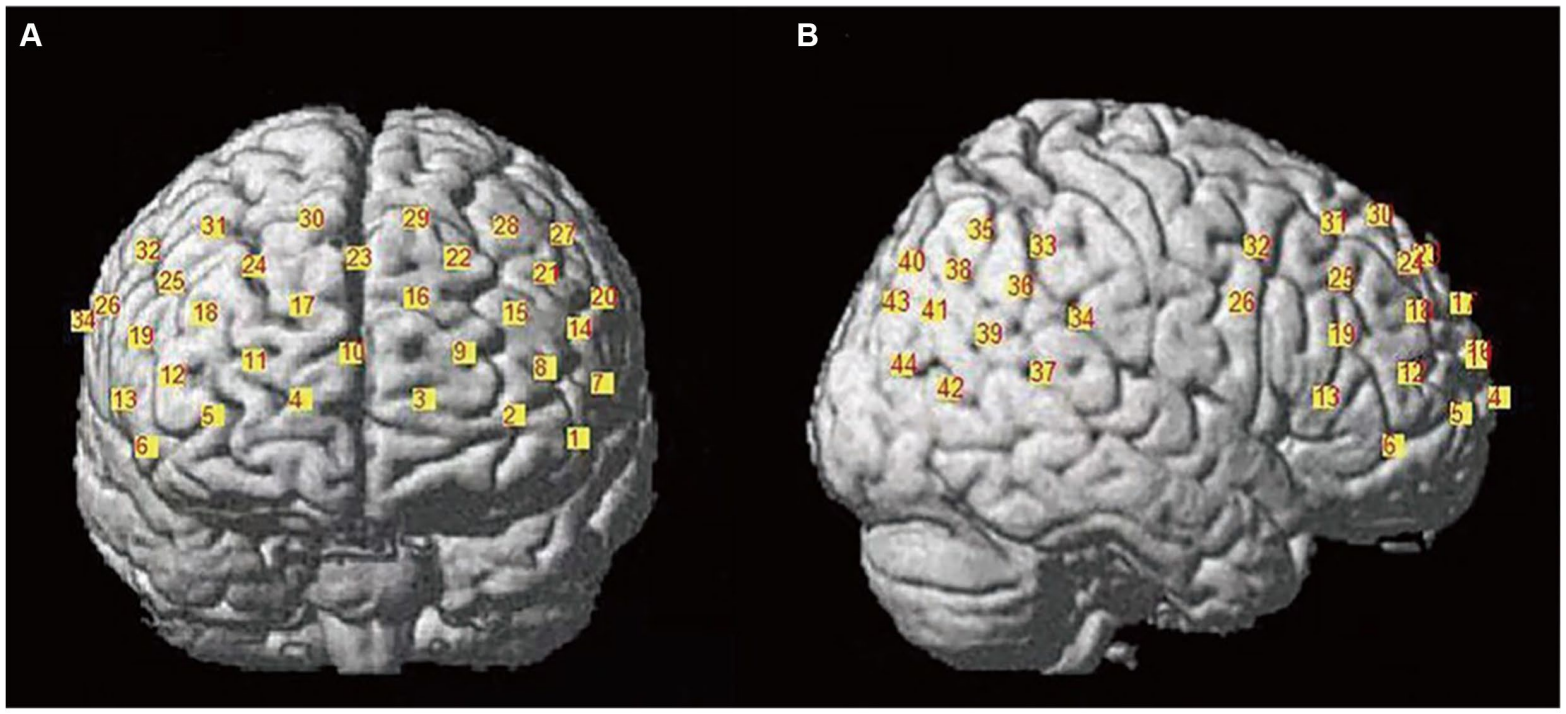
Channel layout diagram. **(A)** The left side shows the prefrontal cortex channel layout, comprising a total of 32 channels; **(B)** the right side shows the temporoparietal junction channel layout, consisting of 12 channels.

The LABNIRS functional near-infrared spectroscopy imaging system (LABNIRS/16, Shimadzu Corporation, Kyoto, Japan) was used. It employs three wavelengths (780, 805, and 830 nm) and the modified Beer–Lambert law (MBLL) to measure changes in oxyhemoglobin (Δ[HbO]), deoxyhemoglobin (Δ[HbR]), and total hemoglobin (Δ[HbT]) concentrations continuously. The sampling rate was 30.303 Hz. After the experiment, a 3D positioning system collected brain positional information. Each participant’s optodes were divided into two sections: the PFC with a 3 × 7 array of 10 emitters and 11 detectors forming 32 channels, and the temporo-parietal junction with a 3 × 3 array of four emitters and five detectors forming 12 channels (see Figure 2). The emitter-detector distance was 3 cm.

### 2.4 Data analysis

#### 2.4.1 Behavioral data analysis

Firstly, descriptive statistics and reliability analyses were conducted on the post-questionnaire results to understand the extent of psychological changes elicited by the experimental manipulation and to establish a foundation for further data selection. Subsequently, a correlation analysis was performed on the post-questionnaires of both groups to examine whether there was a correlation between the psychological changes in the two groups. Finally, independent sample t-tests were employed to assess differences between the IRI scores and the RSQ scores for both groups. This aimed to investigate potential discrepancies in empathy and rejection sensitivity between the two groups.

#### 2.4.2 Neural data analysis

Some studies have indicated a heightened sensitivity of oxyhemoglobin to task stimuli (Hoshi, 2003). Hence, this study exclusively focused on the analysis of Δ[HbO] indicators. Individual raw data underwent processing using NIRS_SPM, operated through Matlab2013a (Ye et al., 2009). The data were processed based on the Hemodynamic Response Functions (HRF) and Wavelet-Minimum Description Length (Wavelet-MDL) method to eliminate noise sources such as head movement and heartbeat, as well as drift. The HRF method was selected to conduct low-pass filtering for high-frequency noise removal. Subsequently, task-related *β*-values for each channel were assessed using the General Linear Model (GLM).

The study primarily focused on the PFC and TPJ. Therefore, The ROIs were delineated for these areas. The PFC encompassed bilateral dorsolateral prefrontal cortex (dlPFC), medial prefrontal cortex (mPFC), and bilateral ventrolateral prefrontal cortex (vlPFC), while the TPJ comprised the right superior temporal gyrus (STG), right angular gyrus (AG), and right supramarginal gyrus (SMG). These corresponded to Brodmann areas as outlined in Table 1. Considering the lower spatial resolution and signal-to-noise ratio of NIRS technology, this study employed individualized *β*-value weighted averaging of channels to transform them into ROIs. Previous research often used averaged channel ROIs, neglecting individualization when converting channel *β*-values to ROIs (Okamoto et al., 2009; Sun et al., 2019). However, this study collected spatial information for all participants’ channels, enabling individualized channel-to-ROI operations based on each individual’s spatial coordinates. Specifically, for example, participant A has channels 5, 7, and 10, with spatial coverage ratios in the medial prefrontal cortex of 60, 70, and 80%, respectively, (to avoid the influence of extreme values, only brain regions with coverage probabilities greater than 50% are taken as channels for merging into specific brain regions). Under the exclusion condition, the likelihood estimates for these three channels are *β*5, *β*7, and *β*10, respectively. Therefore, we calculate the likelihood estimate for participant A’s medial prefrontal cortex under the exclusion condition as:


$$\beta =\left(\beta 5^{*}\;60\%+\beta 7^{*}\;70\%+\beta 10^{*}\;80\%\right)/3.$$

**Table 1:** Correspondence between ROIs and Brodmann area.

ROI	Brodmann area
rSTG	BA22
rSMG	BA40
rAG	BA39
mPFC	BA10
lvlPFC	BA45/BA47
rvlPFC	BA45/BA47
lDLPFC	BA46
rDLPFC	BA46

The inclusion condition is similar. Each ROI for all participants is calculated using this method. Although this approach requires more work, it provides individualized localization information, theoretically offering greater credibility and validity. Then we will conduct a Fisher’s *Z* transformation on the *β* values based on the ROI to obtain standardized *β* values for the subsequent statistical tests.

Subsequently, a repeated measures analysis of variance (ANOVA) was conducted using SPSS software (IBM SPSS Statistics 26.0). The design included factors of 8 (ROIs: right STG, right SMG, right AG, mPFC, left vlPFC, right vlPFC, left dlPFC, right dlPFC) × 2 (conditions: inclusion, exclusion) × 2 (gender: male, female). Corrections for degrees of freedom were applied using the Greenhouse–Geisser method, and post-hoc multiple comparison corrections were conducted using the Bonferroni method.

#### 2.4.3 Neuro-behavioral correlation analysis

We conducted a correlation analysis between brain activation during exclusion compared to inclusion and behavioral data. Specifically, we calculated the standardized *β* values difference (exclusion condition minus inclusion condition) for the eight ROIs in both Targets and Observers. We then performed Pearson correlation analysis between the exclusion–inclusion increment and IRI-C scores as well as RSQ scores. Finally, we applied FDR correction to the results based on the number of ROIs.

## 3 Results

### 3.1 Behavioral data results

The post-questionnaire for targets yielded a Cronbach’s α coefficient of 0.88. Targets self-assessed their average probability of receiving the ball at 23.51%. A one-sample t-test comparing the observed ball receptions by targets (33.3%) against an expected rate revealed significant awareness of receiving fewer passes, *t*(34) = −5.79, *p* < 0.0001, Cohen’s *d* = 0.98. Moreover, descriptive statistics were conducted on the Need Threat Scale, showing an overall mean score of 56.29, indicating a range between ‘rarely (60)’ and ‘somewhat true (40)’. This suggests that targets experienced exclusion during the experiment, indicating the effectiveness of our experimental manipulation.

The post-questionnaire for observers exhibited a Cronbach’s *α* coefficient of 0.76. Descriptive analysis of the questionnaire revealed that the average empathy score for observers was 25.83, which falls within the range of ‘somewhat appropriate (20)’ to ‘reasonably appropriate (30)’. This suggests that observers developed empathy toward the targets during the experimental process.

Correlation analysis was conducted between the post-questionnaire results of targets and observers, revealing a non-significant correlation (*r* = −0.14, *p* = 0.41). An independent sample t-test was performed on the IRI-C and RSQ scores of both groups. Observers’ rejection sensitivity (the mean score = 19.70) was notably higher than that of targets (the mean score = 16.66), *t*(78) = −2.67, *p* < 0.01, Cohen’s *d* = 0.64.

### 3.2 Neural data results

#### 3.2.1 Targets results

The Greenhouse–Geisser correction did not yield significant results; therefore, we referred to the multivariate test results for a more robust assessment. There was a significant interaction between the condition and ROIs, *F*(7, 27) = 2.78, *p* = 0.026, *η_p_*^2^ = 0.418. Simple effects analysis revealed that within the mPFC, activation was greater under the exclusion condition compared to the inclusion condition, *F*(1, 33) = 5.15, *p* = 0.03, *η_p_*^2^ = 0.14.

#### 3.2.2 Observers results

**Figure 3:**
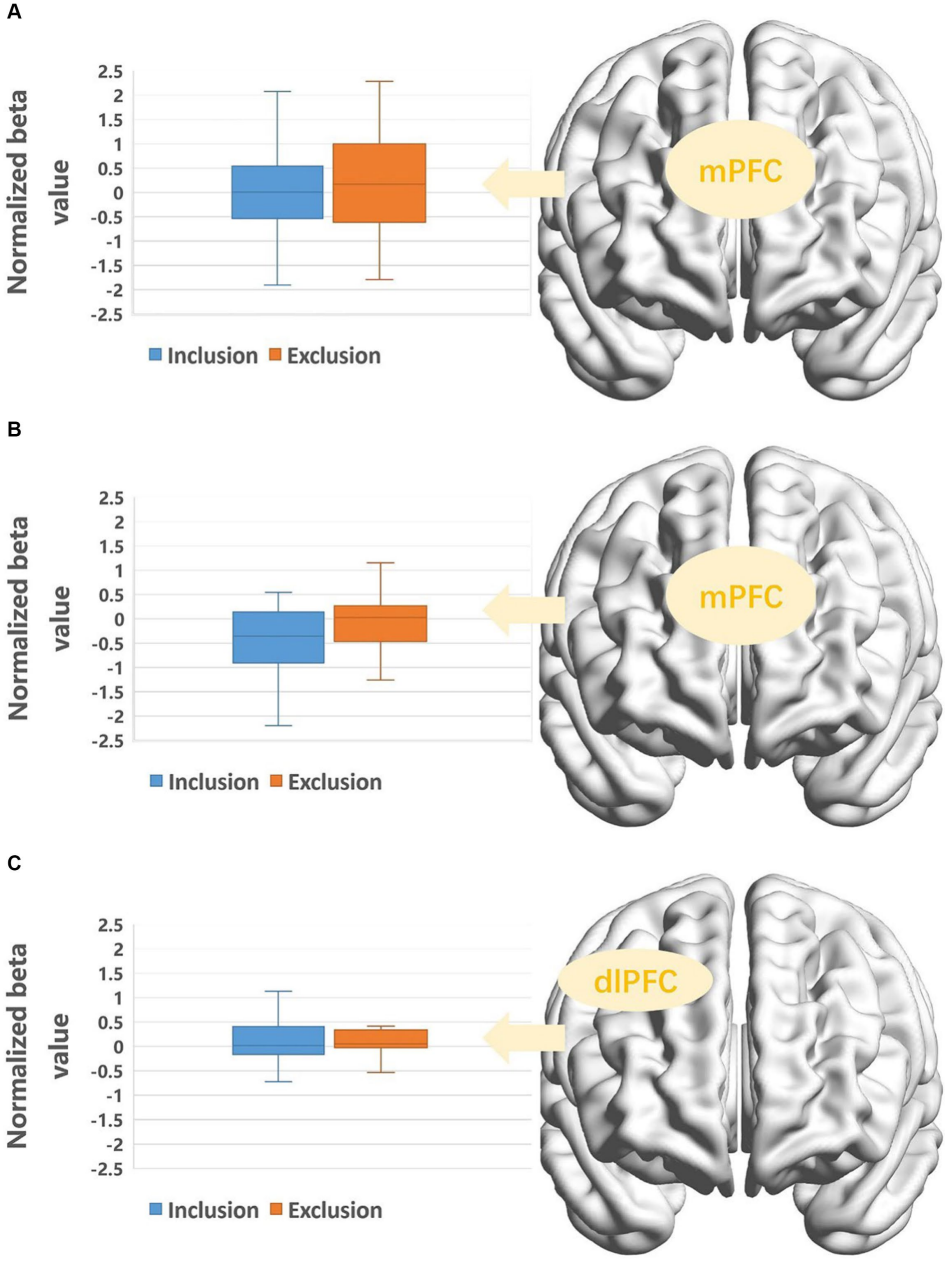
**(A)** Activation of target’s mPFC under different conditions. **(B)** Activation of mPFC in male observers under different conditions. **(C)** Activation of rdlPFC in female observers under different conditions.

An 8 (ROIs: right STG, right SMG, right AG, mPFC, left vlPFC, right vlPFC, left dlPFC, right dlPFC) × 2 (condition: inclusion, exclusion) × 2 (gender: male, female) mixed-measures analysis of variance was conducted to analyze the activation across regions of interest. A significant three-way interaction was found, *F*(7, 27) = 2.77, *p* = 0.039, *η_p_*^2^ = 0.078. Simple effects analyses revealed that in males, the mPFC exhibited higher activation under the exclusion condition compared to the inclusion condition, *F*(1, 33) = 7.19, *p* = 0.011, *η_p_*^2^ = 0.18. Additionally, in females, the right dlPFC showed greater activation under the exclusion condition compared to the inclusion condition, *F*(1, 33) = 4.19, *p* = 0.049, *η_p_*^2^ = 0.11. All significant results of neural activation differences are presented in Figure 3.

### 3.3 Neuro-behavioral results

**Figure 4:**
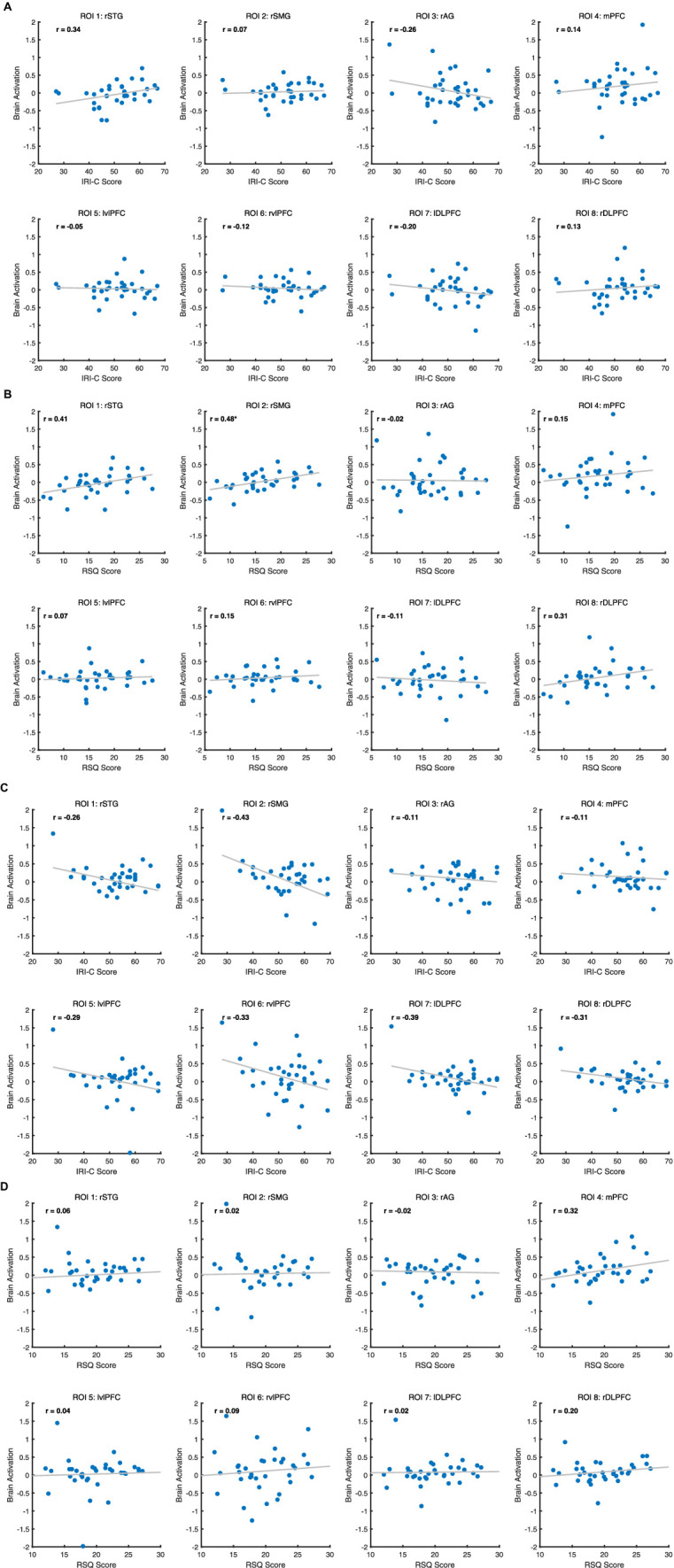
**(A)** Correlation between ROIs and IRI-C score for Target. **(B)** Correlation between ROIs and RSQ score for Target. **(C)** Correlation between eight ROIs and IRI-C score for Observer. **(D)** Correlation between eight ROIs and RSQ score for Observer.

After FDR correction, we found a positive correlation between rSMG brain activation and RSQ scores in the target group (*r* = 0.48, *p* < 0.05). This means that the higher the rejection sensitivity of the participants, the stronger the rSMG activation during exclusion. Other neuro-behavioral correlation results were not significant; detailed results can be found in Figure 4.

## 4 Discussion

Social exclusion stands as a crucial area in the study of emotion generation and development. In order to simulate more authentic responses of individuals within scenarios of social exclusion and to compare the neural mechanisms between vicarious experience and vicarious exposure to social exclusion, this study employs fNIRS technology to concurrently measure the PFC and TPJ of both targets and observers. Additionally, we investigate whether gender influences psychological and neural response patterns. At the behavioral level, it was found that targets and observers exhibited differences in levels of rejection sensitivity following experiences of social exclusion. The extent of exclusion experienced by targets did not correlate with the empathy observed in observers. In terms of neural mechanisms, compared to inclusion blocks, targets exhibited significant activation in the mPFC when exposed to exclusion blocks. Moreover, observers showed gender-specific differences in neural activation patterns: males, similar to targets, displayed increased activity in the mPFC during exclusion blocks compared to inclusion blocks, whereas females exhibited heightened activation in the right dlPFC during exclusion blocks. Finally, we found that the increase in rSMG activation in Targets during exclusion was significantly positively correlated with their rejection sensitivity.

The finding that targets exhibit lower rejection sensitivity compared to observers is noteworthy. Rejection sensitivity reflects an individual’s anxiety about anticipating negative feedback, their tendency to perceive rejection, and their propensity to overreact (Downey and Feldman, 1996). It encompasses emotional and psychological responses to rejection, denial, or non-acceptance, and is influenced by factors such as gender, appearance, personality, early trauma, attachment styles, parenting, peer relationships, and cultural differences (Qinyi et al., 2019). In this study, both targets and observers underwent social exclusion, but targets experienced it directly while observers experienced it vicariously. We hypothesize that direct experience may lead to temporary emotional numbing. Baumeister et al. (2009) suggest that social exclusion can induce emotional and physiological numbing, making individuals less sensitive to emotional stimuli and leading to delayed reactions. This numbing helps in reallocating cognitive resources away from painful information, similar to how animals release opioid-like substances to mitigate pain (Baumeister et al., 2009). Thus, compared to vicarious observers, those who directly experience social exclusion show greater emotional numbness and lower rejection sensitivity.

The sense of exclusion in targets does not correlate with the empathy level of observers, potentially due to the social pain cues evoked by social exclusion, which are significantly influenced by individual experiences compared to cues from physical pain. In a study on social pain empathy, it was found that individuals who vicarious experienced social exclusion tend to underestimate the severity of social pain felt by targets, unless they genuinely comprehend the extent of social exclusion, such as through vicarious experience (Nordgren et al., 2011). Reviews on the effects of vicarious social exclusion suggest that it is impacted by the social relationship, familiarity, and similarity between observers and targets (Singer and Lamm, 2009). Consequently, unfamiliarity, lack of familiarity, and dissimilarity in social relationships diminish the extent to which basic needs of individuals experiencing vicarious exclusion are obstructed (Giesen and Echterhoff, 2018). The perception of rejection by targets is also influenced by individual personality factors and early experiences. Hence, each individual exhibits varying degrees of perceived threat when faced with exclusion triggered by the Cyberball paradigm. In summary, the empathetic resonance to social pain is not as straightforward as physical pain and is contingent upon the perceptions of both targets and observers.

Targets experiencing social exclusion show increased activation in the mPFC compared to experiencing social inclusion, akin to findings in previous related studies (Moor et al., 2012; Vijayakumar et al., 2017; Wasylyshyn et al., 2018). The mPFC, as part of the social monitoring system, is considered a crucial structure in dynamic exclusion processes (Kawamoto et al., 2015). Studies have revealed that individuals with strong secure attachment exhibit weaker mPFC activation when faced with exclusion, rendering them less susceptible to experiencing exclusion (Karremans et al., 2011). Additionally, poorer quality of adolescent community relationships is associated with increased mPFC activation in response to social exclusion (Gonzalez et al., 2015). Individuals with specific mental health conditions also demonstrate varied mPFC responses to social exclusion; for example, people with schizophrenia exhibit decreased activity in the medial prefrontal cortex (mPFC) during social exclusion, while individuals diagnosed with borderline personality disorder and those who engage in self-harm show increased mPFC activation during instances of social exclusion (Gradin et al., 2012; Groschwitz et al., 2016; Wrege et al., 2019). The close association between the mPFC and the recognition of social exclusion is evident. In this study, the mPFC of socially excluded targets exhibited significant activation after experiencing social exclusion, compared to the inclusion phase, reiterating the mPFC’s close association with firsthand experiences of social exclusion.

In the group of observers, gender differences in brain region activation were identified, suggesting that men and women exhibit distinct neural activities when observing others experiencing social exclusion. Specifically, significant conditional differences in the mPFC were observed in males, showing stronger activation when witnessing others being excluded compared to included. Conversely, females displayed significant enhancement in right dlPFC activation when observing others facing exclusion. As mentioned earlier, the mPFC is associated with theory of mind and self-processing (Singer et al., 2006), while the right dlPFC plays a significant role in emotional regulation (Eisenberger et al., 2003). These findings suggest that when witnessing others undergoing social exclusion, males engage the brain areas related to theory of mind, while females show more emotional arousal and regulation. Men might approach situations of social exclusion with a more rational perspective, engaging in more top-down processing of contextual information, contemplating the source and reasons behind social exclusion. Conversely, women tend to be more emotionally responsive, displaying greater empathy toward socially excluded targets and triggering emotional resonance (Derntl et al., 2010; Iffland et al., 2014).

Target’s rSMG activation correlates positively with their rejection sensitivity. The rSMG, part of the TPJ, crucially handles social cognition and empathy, affecting interaction with others and processing social cues (Tousignant et al., 2018). This region often activates during social information processing and emotional responses. Rejection sensitivity (RSQ scores) mirrors one’s anticipation and emotional reaction to social exclusion. Higher sensitivity suggests greater emotional vulnerability to rejection, intensifying negative emotions and stress during exclusion. This emotional response likely boosts rSMG activity, engaged in processing such complex social cues. Surprisingly, we found no brain region activation in observers correlating with their IRI-C levels. Observers may favor cognitive over emotional processing of exclusion, focusing on event context and causality, unlike Targets emotionally affected by exclusion. Therefore, their IRI-C levels may be influenced by non-neural factors. Future studies should refine paradigms for a more comprehensive investigation.

The study has some limitations. Firstly, the inability to measure the anterior cingulate cortex (ACC), an important brain region associated with social exclusion, was due to the constraints of fNIRS in detecting deep brain areas. Accommodating deep brain assessments was unfeasible to achieve multi-brain real-time interaction measurements. Secondly, the participant pool predominantly comprised university students, resulting in a narrow sample, raising questions about the generalizability of the findings to a broader population. Lastly, the ROI analysis in this study focused solely on condition differences within the regions of interest, leaving uncertainties about differences in other cortical brain regions. Future research could involve comprehensive measurements and comparisons across the entire cortical area within this paradigm.

In conclusion, the study yields the following conclusions: Experiencing social exclusion directly versus vicarious evokes different levels of rejection sensitivity. The experienced feelings after personal social exclusion are not directly related to the empathy level experienced following vicarious social exclusion. Direct and vicarious experiences of social exclusion result in distinct patterns of brain activation, and gender influences the expression of these patterns.

## Data Availability

The original contributions presented in the study are included in the article/supplementary material, further inquiries can be directed to the corresponding author.
