# Supplementary material for: Social exclusion: differences in neural mechanisms underlying direct versus vicarious experience
Source: Front Psychol. 2024 Aug 27;15:1368214. doi: 10.3389/fpsyg.2024.1368214 (PMC11385857; doi:10.3389/fpsyg.2024.1368214)
Supplement: Supplementary file 1 [file Data_Sheet_1.docx]

Post-Pass Game Observer Empathy Level Assessment Questionnaire

Please carefully read the following sentence. Choose the appropriate numerical expression based on how well it corresponds to your actual situation during phases of the game with fewer passes being made by players.

1. When I see him/her receiving the ball infrequently in a round, I feel sorry for him/her.

0 - Inappropriate

1 - Slightly appropriate

2 - Moderately appropriate

3 - Appropriate

4 - Very appropriate

1. When I watch game rounds where he/she frequently fails to receive the ball, I feel distressed.

0 - Inappropriate

1 - Slightly appropriate

2 - Moderately appropriate

3 - Appropriate

4 - Very appropriate

1. When I see him/her always failing to receive the ball, I feel like caring for him/her.

0 - Inappropriate

1 - Slightly appropriate

2 - Moderately appropriate

3 - Appropriate

4 - Very appropriate

1. When I see him/her constantly unable to get the ball, I don't imagine how I would feel if I were him/her.

0 - Inappropriate

1 - Slightly appropriate

2 - Moderately appropriate

3 - Appropriate

4 - Very appropriate

1. When watching the game, I don't consider how the players in the game perceive the passing process.

0 - Inappropriate

1 - Slightly appropriate

2 - Moderately appropriate

3 - Appropriate

4 - Very appropriate

1. When I see him/her consistently failing to receive the ball, I don't sympathize with him/her.

0 - Inappropriate

1 - Slightly appropriate

2 - Moderately appropriate

3 - Appropriate

4 - Very appropriate

1. When watching the game, I try to see the passing process from his/her perspective.

0 - Inappropriate

1 - Slightly appropriate

2 - Moderately appropriate

3 - Appropriate

4 - Very appropriate

1. When I see him/her being treated unfairly, I feel sympathy for him/her.

0 - Inappropriate

1 - Slightly appropriate

2 - Moderately appropriate

3 - Appropriate

4 - Very appropriate

1. When I see him/her being treated unfairly, I don't feel sorry for him/her.

0 - Inappropriate

1 - Slightly appropriate

2 - Moderately appropriate

3 - Appropriate

4 - Very appropriate

1. When I see him/her constantly unable to get the ball, I try to imagine how I would feel if I were him/her.

0 - Inappropriate

1 - Slightly appropriate

2 - Moderately appropriate

3 - Appropriate

4 - Very appropriate

The questionnaire comprises ten questions. The initial two questions are adapted from Masten et al.'s research (Masten et al., 2011), while the subsequent eight questions are modeled after the "Empathy concern" and "Perspective taking" subscales of the Interpersonal Reactivity Index (IRI) (Davis, 1980, 1983; Fengfeng et al., 2010). These questions gauge whether observers experienced empathy towards the subjects within the same dyad in the context of social pain.

Scoring is based on a scale of 0-4 points for each item. Items 4, 5, 6, and 9 are reverse-scored, while the remainder are scored positively. A higher total score indicates stronger empathy from the observer towards the subject. In this study, the Cronbach's α coefficient is 0.76.

Reference

Davis, M. H. (1980). A multidimensional approach to individual differences in empathy. *JSAS Catalog of Selected Documents in Psychology*, *10*, 85.

Davis, M. H. (1983). Measuring individual differences in empathy: Evidence for a multidimensional approach. *Journal of Personality and Social Psychology*, *44*(1), 113-126.

Fengfeng, Z., Yi, D., Kai, W., Zhiyu, Z., & Lunfang, X. (2010). Reliability and Validity of the Chinese Version of the Interpersonal Reactivity Index-C. *Chinese Journal of Clinical Psychology*, *18*(2), 155-157.

Masten, C. L., Morelli, S. A., & Eisenberger, N. I. (2011). An fMRI investigation of empathy for 'social pain' and subsequent prosocial behavior. *Neuroimage*, *55*(1), 381-388.
